# Effects of plant growth regulators on transient expression of foreign gene in *Nicotiana benthamiana* L. leaves

**DOI:** 10.1186/s40643-021-00480-5

**Published:** 2021-12-14

**Authors:** Ying Li, Min Sun, Xin Wang, Yue-Jing Zhang, Xiao-Wei Da, Ling-Yun Jia, Hai-Long Pang, Han-Qing Feng

**Affiliations:** School of Life Sciences, Northwest Normal University, Lanzhou, 730070 China

**Keywords:** Plant growth regulators, *Agrobacterium*-mediated transformation, Geminivirus-derived vector, Transient expression

## Abstract

**Background:**

In the last decades, replicating expression vectors based on plant geminivirus have been widely used for enhancing the efficiency of plant transient expression. By using the replicating expression vector derived from bean yellow dwarf virus and green fluorescent protein as a reporter, we investigated the effects of α-naphthalene acetic acid, gibberellins_3_, and 6-benzyladenine, as three common plant growth regulators, on the plant biomass and efficiency of transient expression during the process of transient expression in *Nicotiana benthamiana* L. leaves.

**Results:**

With the increase of the concentration of α-naphthalene acetic acid, gibberellins_3_, and 6-benzyladenine (from 0.1 to 1.6 mg/L), the fresh weight, dry weight, and leaf area of the seedlings increased first and then returned to the levels similar to the controls (without chemical treatment). The treatment with α-naphthalene acetic acid at 0.2 and 0.4 mg/L can enhance the level of transient expression of green fluorescent protein, which peaked at 0.4 mg/L α-naphthalene acetic acid and was increased about by 19%, compared to the controls. Gibberellins_3_ at 0.1–0.4 mg/L can enhance the level of transient expression of green fluorescent protein, which peaked at 0.2 mg/L gibberellins_3_ and was increased by 25%. However, the application of 6-benzyladenine led to decrease in the level of transient expression of green fluorescent protein.

**Conclusions:**

The appropriate plant growth regulators at moderate concentration could be beneficial to the expression of foreign genes from the *Agrobacterium*-mediated transient expression system in plants. Thus, appropriate plant growth regulators could be considered as exogenous components that are applied for the production of recombinant protein by plant-based transient expression systems.

**Supplementary Information:**

The online version contains supplementary material available at 10.1186/s40643-021-00480-5.

## Introduction

Transient expression technology refers to the technology of introducing the target gene into recipient cells to establish a temporary high-efficiency expression system so that the target gene can be expressed in a relatively short time. The transformed target gene by the transient expression technology is not integrated into the genome, thus saving the time of genetic transformation and screening (Chen et al. 2013).

Production of the desired protein via transient expression has obvious advantages, including less time with more protein expression, lower cost, and easy manipulation without any biosafety concerns, compared to develop stable transgenic lines with time-consuming procedures (Xia et al. 2020). And, in recent years, plants have been emerged as an alternative platform for the production of recombinant proteins to meet the worldwide demand for protein-based pharmaceuticals (Chen 2008). Compared to animal, yeast, and bacteria cells, the production of recombinant proteins by plant cells is more reliable, scalable, low cost, and safe (Damaj et al. 2020). And, the recombinant proteins that are produced by transient expression in plants can be extracted within 1–2 weeks after transformation (Leuzinger et al. 2013). Especially today, when new pathogens and diseases are prone to sudden outbreaks, the production of medicinal proteins or antibodies in plants by transient expression system is of great significance for social security and disease treatment (Peyret and Lomonossoff 2015).

For the transient expression of foreign gene in plant, the target gene is transferred into plant cells through a process called agroinfiltration. In simply, leaves are infiltrated with the suspension of *Agrobacterium tumefaciens* carrying the target gene within a binary vector. Infection of *Agrobacterium tumefaciens* delivers the target gene into the plant cells. After entering into the plant cells, the most of the target gene remains episomal, instead of integrating into the genome, and can have transcriptionally competent during certain time (Leuzinger et al. 2013).

*Nicotiana benthamiana*, as a wild relative of tobacco, has been considered as model organism to study the plant-based transient expression. The main reasons underlying this is that *Nicotiana benthamiana* is a popular host for *Agrobacterium tumefaciens*, and thus, *Nicotiana benthamiana* is highly amenable to *Agrobacterium*-mediated transient expression (Reed and Osbourn 2018).

In the last decades, autonomously replicating expression vectors based on plant geminivirus has been widely used in plant transient expression (Hefferon 2014; Rybicki and Martin 2014). Compared with those non-replicating expression vectors, autonomously replicating expression vectors based on plant geminivirus can produce a large number of copies of target genes after transformation, thus largely enhancing the expression level of the desired protein (Abrahamian et al. 2020). For example, the bean yellow dwarf virus (BeYDV) is one of the geminiviruses in the genus Megaviruses. Its genome is circular single-stranded DNA with a size of 2561 bp. After infecting the host plant, BeYDV quickly replicates to a high copy number in the host cell nucleus by rolling circle replication (Chen et al. 2011; Zaidi and Mansoor 2017). Based on the above characteristics, the BeYDV-derived expression vectors are constructed, which contain the replication-related elements of BeYDV and an expression cassette for a gene of interest (Zhang and Mason 2006; Baltes et al. 2014). Many works have shown that using the BeYDV-derived expression vectors in plant transient expression can generate a large number of target gene copies in plant host cells, thus greatly improving the efficiency of plant transient expression (Richter et al. 2016; Hanley-Bowdoin et al. 2013).

Besides the development of new expression vectors, some efforts are also attempted to further improve the efficiency of transient gene expression in plants. These include the optimization of internal or external factors that could affect the efficiency of plant transient expression, such as the concentration of *Agrobacterium tumefaciens*, temperature, light intensity, and humidity (Maleki et al. 2018; Fujiuchi et al. 2016). Although some internal factors can be easily controlled, it is still difficult to obtain higher protein yield from transient expression in plants by modifying external environmental factors, especially when the plants used for transient expression were grown in the field. Thus, developing some novel methods that can be easily applied is still needed for enhancing the utility of the plant-based transient expression systems.

Plant growth regulators (PGRs) are a kind of chemical substances synthesized artificially, which can regulate the growth and development of plants by exogenous application (Gong et al. 2021). α-naphthalene acetic acid (NAA), gibberellins_3_ (GA_3_), and 6-Benzyladenine (6-BA) are commonly used in agriculture for enhancing the production of crops. NAA, which belongs to the synthetic branch of auxins, is a naphthalene derivative widely used to stimulate plant growth, prevent the premature fall of fruits, and increase the yield of crops (Guan et al. 2011). GA_3_ is a dihydroxylated gibberellin and can promote the growth and development of plants. GA_3_ has been applied to regulate seed germination, organ elongation, flowering, and fruit maturity by affecting mitotic frequency or cell enlargement (Hedden and Sponsel 2015). 6-BA is a synthetic cytokinin, which can mediate various physiological processes of plants, such as plant stem and root growth, cell proliferation, chloroplast development, and biomass distribution (Werner et al. 2010; Hwang et al. 2012), thus severing as the regulator for promoting plant growth, increasing chlorophyll content, and delaying senescence (To and Kieber 2008). More importantly, these PGRs are lower cost, relatively cheap, and are easy to apply in the field. However, information about the effects of PGRs on the efficiency of plant transient expression is very limited.

Therefore, in this study, by using the BeYDV-derived replicating expression vector and green fluorescent protein (GFP) as a reporter, we investigated the effects of NAA, GA_3_, and 6-BA, as three common PGRs, on the plant biomass and efficiency of transient expression during the process of transformation in *Nicotiana benthamiana* L. leaves. We believe that this study would help provide a reference for how to utilize PGRs to improve the yields of recombinant protein from plant transient expression.

## Materials and methods

### Experimental material

#### Cultivation of plant materials

Two or three seeds of *Nicotiana benthamiana* L. were planted into the peat pell in the seedling box and were cultured in a culture room with a temperature of 25 ℃, a humidity of 50%, and a day and night period of 16/8 h. Hoagland nutrient solution was applied for seedling growth. The 3-week-old seedlings were moved from the seedling box to provide more sufficient space for further growth until they were ready to be treated at the 4th week of growth.

#### The geminivirus-derived expression vector and ***Agrobacterium*** transformation

The expression vector based on BeYDV was presented by Professor Mason of Arizona State University. GFP gene was constructed downstream of the 35S promoter in the vector as the reporter gene. The expression vector based on BeYDV was transformed into this *Agrobacterium* LBA4404 strain, and the antibiotic resistance gene of the vector was used for screening and subculture.

#### Preparation of *Agrobacterium* cultures

*Agrobacterium tumefaciens* LBA4404 strain harboring the BeYDV-derived expression vector with the GFP gene was streaked on *Agrobacterium* rhizogene medium (YEB) Agar plates containing kanamycin (50 μg/ml), rifampicin (25 μg/ml), and chloramphenicol (25 μg/ml). After 24 h of growth, the monoclone was picked out from the YEB agar plate and inoculated into 50 mL YEB broth liquid medium with the antibiotics at 28 °C overnight in a shaker at 180 rpm. The bacteria were collected by centrifugation at 5000 rpm for 10 min and then washed by infiltration buffer (10 mM MES-KOH, pH 5.5; 10 mM MgSO4; 100 μM Acetosyringone). After then, the bacteria were collected again and re-suspended in the infiltration buffer. The bacterial concentrations were determined by measuring optical density (OD) at 600 nm and were diluted to OD_600_ 0.3 with the infiltration buffer.

#### PGRs treatment and plant transformation

Three PGRs, NAA, GA_3_, or 6-BA, with different concentrations (0.1, 0.2, 0.4, 0.8, and 1.6 mg/L, respectively), were prepared with distilled deionized water. The seedlings were sprayed three times with different concentrations of NAA, GA_3_, or 6-BA at 3 days of interval (i.e., for the first spraying, the leaves of 4-week-old seedlings were sprayed with different concentrations of NAA, GA_3_, or 6-BA; the second spraying was performed at 3 days after the first spraying, and the third spraying was performed at 3 days after the second spraying). In the control group, the leaves of the seedlings were sprayed with the same amount of distilled deionized water at the under time and under the same conditions. Each spray application was performed until the leaf was wet and the solution ran off. Isolation barriers were set in each spraying to avoid cross-influence.

At 9 days after the first spraying with PGRs, the growth of the sprayed plants was observed, and the parameters of fresh weight, dry weight, and leaf area were measured. At 9 days after the first spraying with PGRs, a small gap was slightly cut in the lower epidermis along the main vein of the leaf, which is 1/3 away from the leaf base and 0.5 cm away from the main vein. Two mL of *Agrobacterium* suspension was slowly injected into the leaf through the gap with a sterile syringe without a needle. After infiltration, the plants were moved back to the culture room, and the expression of GFP was monitored at the 4th day after infiltration.

#### Determination of biomass

An analytical balance was used to measure the fresh weight of the aerial part of the seedlings. After then, the aerial part of seedlings was put in an oven for deactivation of enzymes at 100 ℃ for 20 min, dried to constant weight at 70 ℃, and then taken out for weighing the dry weight. For the determination of leaf area, photos of all leaves on each seedling were taken by camera, and Photoshop software was used to measure the leaf area.

#### Detection of GFP expression

At the 4th day after the injection of the infiltration buffer, the lower surface of the infiltrated leaf was placed under a Leica fluorescent stereomicroscope (Leica Microsystems Ltd. DFC450 C). A region of 1 cm away from the original injection site was selected. This region was excited at a wavelength of 450–490 nm and the emission spectrum between 500 and 550 nm was recorded.

#### Data analysis

The leaf area was measured by Photoshop CS5 software, the GFP fluorescence was analyzed by Image J software, and the data were analyzed by one-way ANOVA with IBM SPSS Statistics 19 software. LSD and Duncan methods were used for multiple comparisons and significance analysis, and the significant difference was expressed by *P*  < 0.05. The value obtained is the average value of at least 3 independent experiments, and the data are expressed as mean value standard error (SE).

## Results

### Effect of NAA on growth and transient expression of *Nicotiana benthamiana* L. seedlings

In this study, strong GFP expression was observed in the leaves that were infected with LBA4404 carrying GFP gene in the BeYDV-derived expression vector, while green fluorescence was not observed in the leaves infected by the *Agrobacterium* LBA4404 strain without expression vector (Additional file 1: Fig. S1). Thus, the observed green fluorescence was specifically from the expression of the GFP gene.

**Fig. 1:**
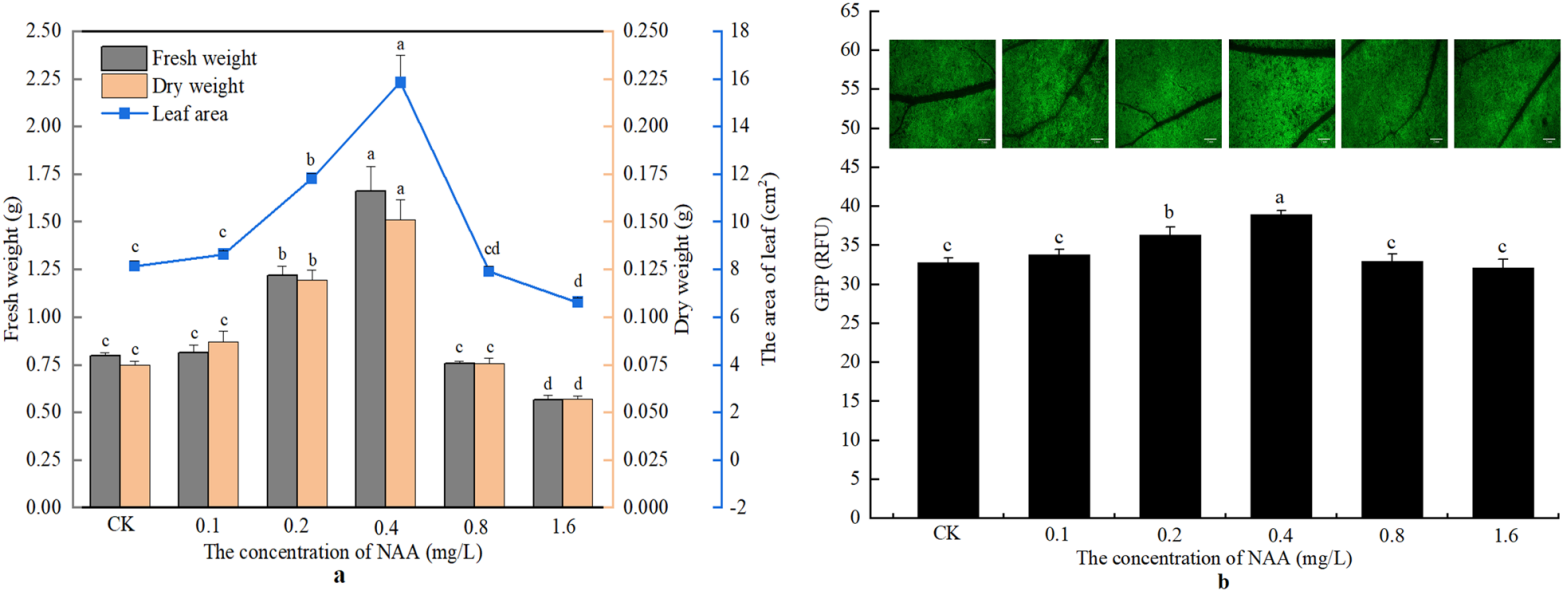
The changes in biomass and GFP fluorescence intensity of seedlings under different NAA concentrations. **a** The changes of fresh weight, dry weight, and leaf area of the seedlings after treatment with different concentrations of NAA. **b** The changes in GFP fluorescence intensity in the leaves of the seedlings treated with different concentrations of NAA. Bar  = 2 mm. CK, Treatment with water; NAA, α-naphthalene acetic acid. The values of RFU (relative fluorescence unit) are average  ±  standard error (SE) of three individual replicates. Different lowercase letters indicate that there are significant differences in the same parameter among the treatment with different concentrations of NAA at the *P*  < 0.05 level

It can be observed from Fig. 1a that the treatment with 0.1 mg/L NAA did not significantly affect the biomass of the seedlings, compared to the control (treatment with water). NAA at 0.2 or 0.4 mg/L significantly increased the fresh weight, dry weight, and leaf area of the seedlings, and the biomass of the seedlings treated with 0.4 mg/L NAA was significantly higher than that of the seedlings treated with 0.2 mg/L NAA. However, the 0.8 mg/L NAA failed to increase the fresh weight, dry weight, and leaf area of the seedlings. When the concentration of NAA reached 1.6 mg/L, the biomass of the seedlings presented a decrease, compared with the control. The fresh weight and dry weight of the seedlings treated with 1.6 mg/L NAA was decreased significantly by 29.12% and 23.54%, respectively, while the leaf area was decreased significantly by 18.56%, compared with the control.

The change of the green fluorescence from GFP expression in the infected leaves showed the same trend as that of the biomass of the seedlings (Fig. 1b). Compared with the control, the expression of GFP in the seedlings increased by 3.12%, 10.91%, 19.00%, 0.57%, and − 1.92% after treatment with 0.1, 0.2, 0.4, 0.8, and 1.6 mg/L of NAA, respectively.

### Effect of GA_3_ on growth and the transient expression of the ***Nicotiana benthamiana*** L. seedlings

**Fig. 2:**
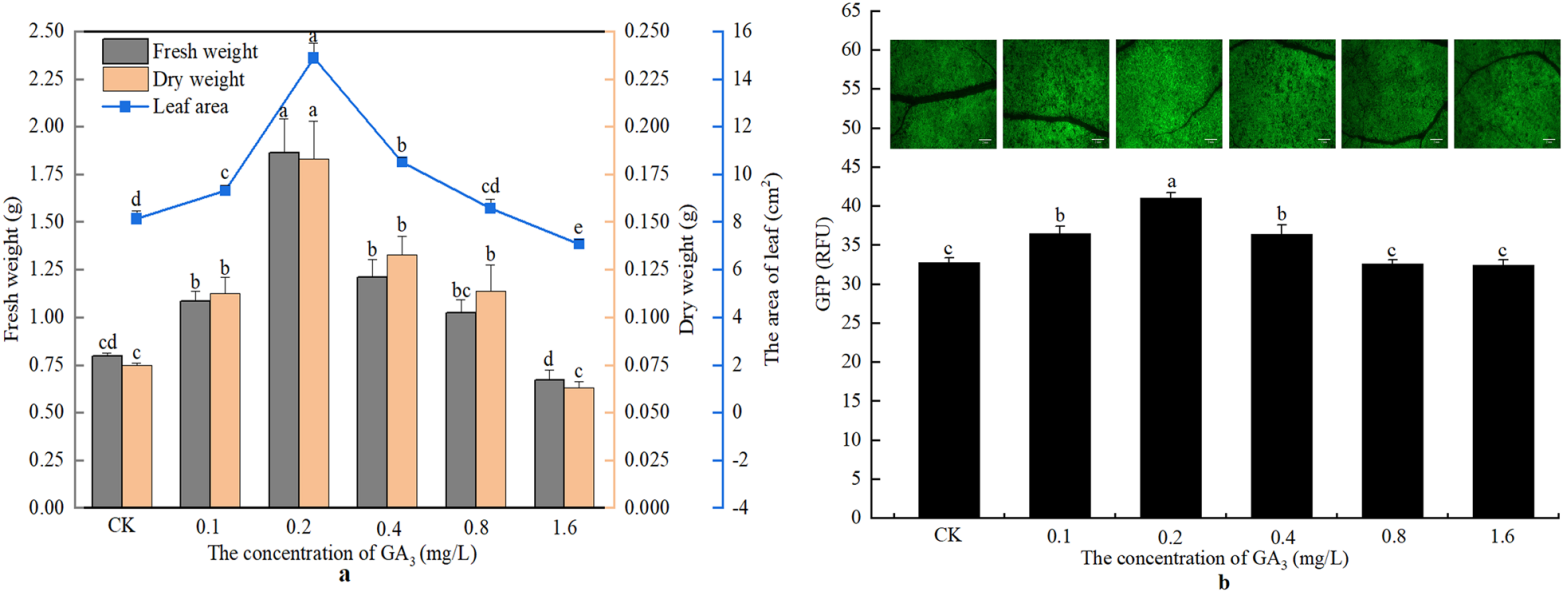
The changes in biomass and GFP fluorescence intensity of seedlings under different GA_3_ concentrations. **a** The changes of fresh weight, dry weight, and leaf area of the seedlings after treatment with different concentrations of GA_3_. **b** The changes in GFP fluorescence intensity in the leaves of the seedlings treated with different concentrations of GA_3_. Bar  = 2 mm. CK, Treatment with water; GA_3_: gibberellins_3_. The values of RFU (relative fluorescence unit) are average  ±  standard error (SE) of three individual replicates. Different lowercase letters indicate that there are significant differences in the same parameter among the treatment with different concentrations of GA3 at the *P*  <  0.05 level

Compared with the control, the treatment with 0.1–0.4 mg/L GA_3_ significantly increased the levels of all of the biomass parameters measured (including fresh weight, dry weight, and leaf area) of the seedlings, which peaked at 0.2 mg/L GA_3_ (Fig. 2a). However, 0.8 mg/L GA_3_ failed to increase the fresh weight and leaf area of the seedlings but increased the dry weight to some extent. When the concentration of GA_3_ reached 1.6 mg/L, the biomass of the seedlings returned to a level similar to that in the controls.

With the increase of GA_3_ concentration, the expression of GFP increased at first and then decreased (Fig. 2b). Similar to the effects of GA_3_ on plant growth, 0.1–0.4 mg/L GA_3_ significantly increased the expression of GFP in the seedlings. The enhancement of GFP expression is most obvious when 0.2 mg/L GA_3_ was used (increase by 25.28%, compared to the controls). GA_3_ at other concentration had no significant effects on GFP expression.

### Effect of 6-BA on growth and on transient expression of the *Nicotiana benthamiana* L. seedlings

**Fig. 3:**
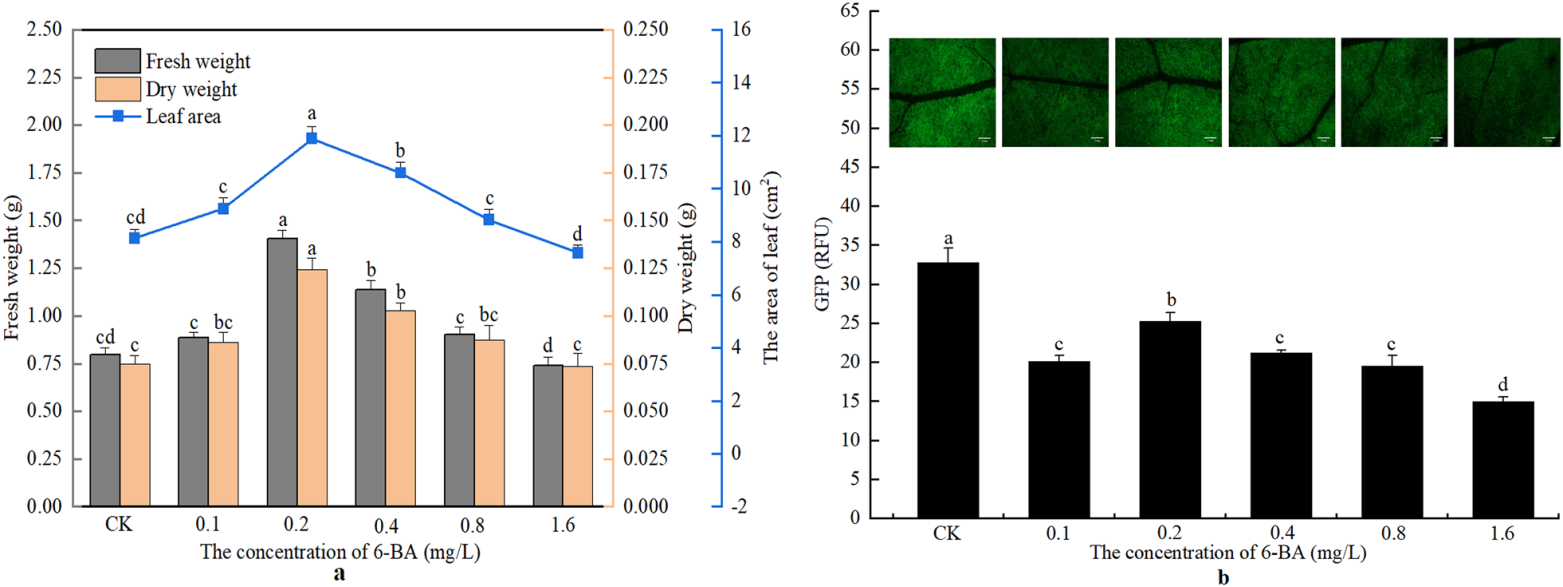
The changes in biomass and GFP fluorescence intensity of seedlings under different 6-BA concentrations. **a** The changes of fresh weight, dry weight, and leaf area of the seedlings after treatment with different concentrations of 6-BA. **b** The changes in GFP fluorescence intensity in the leaves of the seedlings treated with different concentrations of 6-BA. Bar  = 2 mm. CK, Treatment with water; 6-BA: 6-benzyladenine. The values of RFU (relative fluorescence unit) are average  ±  standard error (SE) of three individual replicates. Different lowercase letters indicate that there are significant differences in the same parameter among the treatment with different concentrations of 6-BA at the *P*  < 0.05 level

Treatment with 6-BA at 0.2 or 0.4 mg/L significantly enhanced the levels of all of the biomass parameters measured of the seedlings, and the enhanced effects of 0.2 mg/L 6-BA on the growth of seedlings was more obvious than 0.4 mg/L 6-BA (Fig. 3a). 6-BA at 0.8 and 1.6 mg/L had no significant effects on the biomass of the seedlings.

Treatment with 6-BA decreased the expression of GFP in the leaves (Fig. 3b). Compared with the control, the expression of GFP decreased by 36.68%, 22.75%, 35.28%, 40.39%, and 54.30%, respectively, after treatment with 0.1, 0.2, 0.4, 0.8, and 1.6 mg/L 6-BA, respectively.

## Discussion

It was not surprising that NAA, GA_3_, and 6-BA at moderate concentration enhanced the plant growth (Figs. 1a;  2a;  3a). In detail, for the enhancement of the growth, the most effective concentration of these three PGRs is different. NAA at 0.4 mg/L led to the most obvious increase in the biomass of the seedlings, while GA_3_ and 6-BA at 0.2 mg/L led to the most obvious increase in the biomass. The discrepancy may originate from the difference in the regulation mechanism of plant growth by these three PGRs. And, our observations are consistent with the previous findings that the effects of auxin, gibberellins, and cytokinin on plant growth are depend on the concentration, with high and low doses eliciting different responses by different mechanisms (Teale et al. 2006; Schaller et al. 2014; Muniandi et al. 2018). For example, below the threshold level, auxin can promote cell division and cell elongation (Wang and Ruan 2013). However, above the threshold level, auxin inhibits cell division and cell elongation. A well-known model of the auxin-stimulated cell expansion is based on the “acid growth theory” (Wang and Ruan 2013). But, the inhibition of cell expansion by high concentration of auxin is attributed to the increase of number of functionally active ARF (auxin response factor) and the transcriptional activation of auxin regulons, rather than the “acid growth theory” (Teale et al. 2006). Thus, the dose–response curve for the growth of plant cells over the increased concentration of exogenous auxins commonly shows the bell-shaped curve (Campanoni and Nick 2005), which was also presented by out observation. In conclusion, a moderate concentration of PGRs for plant growth is needed.

The aim of this study is to evaluate the effects of NAA, GA_3_, and 6-BA on the efficiency of transient expression. The results showed that NAA and GA_3_ at moderate concentration can enhance the level of transient expression. Among the concentrations of NAA used, 0.4 mg/L NAA led to the largest enhancement of transient expression of GFP (transient expression of GFP was enhanced by 19%). For GA_3_, the largest enhancement of transient expression of GFP was achieved by 0.2 mg/L GA_3_ (transient expression of GFP was enhanced by 25%). And, it seems that there was a certain correlation between the transient expression efficiency and growth among the seedlings treated with either NAA or GA_3_ (from Additional file 1: Fig. S2). This raises a possibility that the effect of NAA or GA_3_ on transient expression efficiency of *Nicotiana benthamiana* L. seedlings could be related to the growth of plants. In fact, auxin and gibberellin are known to promote plant growth by inducing cell wall relaxation (Sánchez-Rodríguez et al. 2010). Although loosening the cell wall is a vital process during growth and development, it may also render the plant more vulnerable to biotic intruders by facilitating pathogen entry, allowing enhanced nutrient leakage, and increasing availability of resources for pathogens (Depuydt et al. 2009). Thus, pathogens might benefit from the auxin- or gibberellin-induced enhancement of growth of host. Combining these findings with our observations, we assume that the promoted growth of plants by the moderate concentrations of IAA and GA_3_ is benefit to *Agrobacterium tumefaciens* for their infection and spread. As a result, the transient expression efficiency was increased. With the further increase of concentrations of IAA and GA_3_, the enhanced effect of IAA and GA_3_ on host growth became weaken (or the inhibitory effect of IAA and GA_3_ on host growth increased). This decreased the utilization of host cells by *Agrobacterium tumefaciens* for their infection and spread. This could explain why IAA and GA_3_ did not affect the transient expression efficiency by a dose-dependent manner, and the highest concentrations of IAA and GA_3_ did not effectively enhance the transient expression efficiency.

Different from the effects of IAA and GA_3_ on the transient expression efficiency, the treatment with 6-BA treatment decreased the level of transient expression of GFP, although it can enhance plant growth at a moderate concentration (0.2–0.4 mg/L). It was found that exogenous application of high concentrations (10–100 μm) of 6-BA to Arabidopsis seedlings before pathogen inoculation can decrease the susceptibility of the seedlings to the biotrophic oomycete *Hyaloperonospora arabidopsidis* (Argueso et al. 2012; Albrecht and Argueso 2017). And, some works also showed that cytokinin can induce the resistance of plants to pathogen by salicylic acid-dependent signaling pathway (Albrecht and Argueso 2017). These evidences indicate that 6-BA can actually increase the resistance of the plant to pathogen by directly stimulating host defense. Thus, it is reasonable to assume that 6-BA application could trigger the defense of *Nicotiana benthamiana* L. seedlings to *Agrobacterium tumefaciens*, thus reducing the transient expression efficiency. Such effect could also overcome the positive effects of 6-BA on *Agrobacterium tumefaciens* infection and spread when 6-BA at moderate concentration enhances the growth of host plant.

It should be also noted that the *Agrobacterium*-mediated transient expression is a complex process involving a series of biological events, including *Agrobacterium tumefaciens* infection, T-DNA transfer from *Agrobacterium tumefaciens*, protein biosynthesis, and its accumulation in leaf tissue (Gelvin 2003; Jamal et al. 2009; Matsuda et al. 2018). At the same time, the effects of PGRs on plants are also very complex. Thus, more works are needed to obtain clear mechanism for the effects of different PGRs on the level of transient expression.

Regardless of how complex the mechanisms for the effects of these PGRs on the transient expression, the aim of the present work is to present whether the PGRs could be used to enhance the efficiency of *Agrobacterium*-mediated transient expression systems. In the current works about improving the efficiency of transient gene expression in plants, much attention is focused on the optimization of the vector by molecular biological methods. And, some works also made attempts to enhance the efficiency of transient gene expression by modifying external factors, mainly including light, temperature, and humidity (Fujiuchi et al. 2016, 2014, 2017; Matsuda et al. 2017). However, it is difficult or high cost to control temperature, humidity, and illumination when transient gene expression is utilized to produce pharmaceutical-grade recombinant proteins. In comparison, PGRs can be easily applied and cost-efficient if they can be effective to increase recombinant protein yields from transient gene expression in plants. And, there are no security concerns when PGRs are used. As demonstrated by the present work, appropriate PGRs at moderate concentration could be a benefit for the yield of foreign protein and plant growth. Of course, further work is needed to evaluate the effect of PGRs on the production of pharmaceutical protein from *Agrobacterium*-mediated transient expression system in plants.

In conclusion, appropriate PGRs can enhance the efficiency of transient gene expression. Thus, they have the potential to be considered as the exogenous components to be applied in recombinant protein production by plant-based transient expression systems.

### Supplementary Information

**Additional file 1: Figure S1. **The fluorescent expression of GFP in LBA4404 strains without BeYDV-derived expression vector (left) and with LBA4404 strains containing BeYDV expression vector (right). Bar = 2 mm. **Figure S2. **The correlation between fresh weight, dry weight, leaf area, and GFP expression of the seedlings under 0.4 and 0.2 mg/L treatments of NAA (left) and GA_3_ (right), respectively. The asterisks (*) indicate that different parameters are significantly correlated at the P <0.05 level, and the closer the value is to 1, the stronger the correlation.

## Data Availability

The data generated and/or analyzed during this study are available from the corresponding author on reasonable request.

## Abbreviations

BeYDV: Bean yellow dwarf virus
GFP: Green fluorescent protein
NAA: α-Naphthalene acetic acid
GA_3_: Gibberellins_3_
6-BA: 6-Benzyladenine
PGRs: Plant growth regulators
YEB: *Agrobacterium* rhizogene medium
