# Supplementary material for: Effects of plant growth regulators on transient expression of foreign gene in Nicotiana benthamiana L. leaves
Source: Bioresour Bioprocess. 2021 Dec 14;8(1):124. doi: 10.1186/s40643-021-00480-5 (PMC10992099; doi:10.1186/s40643-021-00480-5)
Supplement: Supplementary file 1 — Additional file 1: Figure S1. The fluorescent expression of GFP in LBA4404 strains without BeYDV-derived expression vector (left) and with LBA4404 strains containing BeYDV expression vector (right). Bar = 2 mm. Figure S2. The correlation between fresh weight, dry weight, leaf area, and GFP expression of the seedlings under 0.4 and 0.2 mg/L treatments of NAA (left) and GA3 (right), respectively. The asterisks (*) indicate that different parameters are significantly correlated at the P <0.05 level, and the closer the value is to 1, the stronger the correlation. [file 40643_2021_480_MOESM1_ESM.docx]

**Experiment Additional Diagram:**


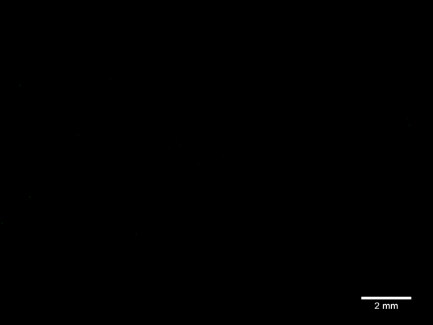


LBA4404 strains without BeYDV-derived expression vector


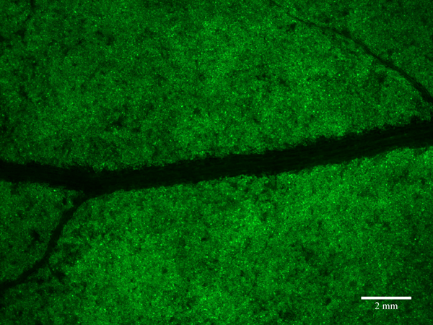


LBA4404 strains containing BeYDV-derived expression vector

**Fig. S1** The fluorescent expression of GFP in LBA4404 strains without BeYDV-derived expression vector (left) and with LBA4404 strains containing BeYDV expression vector (right) Bar = 2 mm.

Correlation between the seedling biomass and GFP through NAA treatment

Correlation between the seedling biomass and GFP through GA_3_ treatment

**Fig. S2** The correlation between fresh weight, dry weight, leaf area, and GFP expression of the seedlings under 0.4 and 0.2 mg/L treatments of NAA (left) and GA_3_ (right), respectively. The asterisks (_*_) indicate that different parameters are significantly correlated at the P <0.05 level, and the closer the value is to 1, the stronger the correlation.
